# An Evaluation of the Temporal Integrator Processing Strategy for Cochlear Implants in Comparison to the Clinical Strategy and in Multi-Talker Noise

**DOI:** 10.1097/AUD.0000000000001741

**Published:** 2025-11-20

**Authors:** Lidea Shahidi, Robert P. Carlyon, Deborah A. Vickers, Tobias Goehring

**Affiliations:** 1 Cambridge Hearing Group, MRC Cognition and Brain Sciences Unit, University of Cambridge, Cambridge, UK; and; 2 Cambridge Hearing Group, Department of Clinical Neurosciences, University of Cambridge, Cambridge, UK.

**Keywords:** Cochlear implant, Speech coding, Speech processing strategy, Temporal masking

## Abstract

**Objectives:**

Speech perception remains challenging for cochlear-implant recipients in conditions containing background noise. The sound processing strategy of the cochlear implant transforms the acoustic signal into a pattern of electrical stimulation pulses, which are transmitted by the electrode array. Modifications to the sound processing strategy could improve the transmission of information by the cochlear implant, alleviating speech perception difficulties in noise. One such modification, the temporal integrator processing strategy (TIPS), uses a model of temporal masking to identify and remove stimulation pulses that are unlikely to be perceived. In a previous investigation, TIPS significantly improved the perception of continuous interleaved sampling-processed speech in the presence of speech-shaped noise while reducing the power required for stimulation (Lamping et al. 2020). This study extends the previous investigation to include conditions that better reflect everyday listening for cochlear-implant recipients.

**Design:**

Two pre-registered double-blind within-participant experiments measured the speech-recognition performance of 12 cochlear implant listeners for sentences before and after TIPS processing. Experiments 1 and 2 investigated TIPS performance against the Continuous Interleaved Sampling or Advanced Combination Encoder baseline speech processing strategies, respectively. Speech-reception thresholds were measured in a speech-shaped noise (in experiment 1) and a multi-talker noise (in experiments 1 and 2), and mean-opinion score speech quality ratings were obtained in quiet (in experiments 1 and 2). In both experiments, all strategies used the subjects’ clinical pulse rate, inter-phase gap, phase duration, and ground electrodes, and participants were acclimatized for 10 min before testing each strategy.

**Results:**

In experiments 1 and 2, the median speech-reception thresholds obtained were comparable across the baseline and TIPS strategies, with no statistical differences noted in either speech-shaped or multi-talker noise. Quality ratings were lower for TIPS when compared to the Continuous Interleaved Sampling strategy (*p* = 0.044) but not when compared to the Advanced Combination Encoder strategy, which all participants used as their everyday strategy. In all conditions, TIPS could reduce device power consumption by between 21% and 42%.

**Conclusions:**

TIPS could result in substantial power savings, without compromising speech quality or speech intelligibility in noise. Although some individuals demonstrated improved speech-reception thresholds with TIPS, the significant group-level improvement previously found by Lamping et al. (2020) was not observed in this study.

## INTRODUCTION

Cochlear implants (CIs) transmit sound by electrically stimulating the auditory nerve. Contemporary CI sound processing strategies are loosely based on continuous interleaved sampling (CIS, Wilson et al. 1991), which encodes envelope information from a discrete range of frequencies in each electrode channel. Although conventional speech processing strategies convey sufficient information about the speech envelope to enable communication for most recipients (Skinner et al. 2002; Firszt et al. 2004), this information is often degraded when background noise is present in the listening environment, worsening communication outcomes (Cullington & Zeng 2008; Dorman & Gifford 2017; Carlyon & Goehring 2021).

One major factor contributing to poorer speech perception outcomes is the spread of electric current along the conductive fluid and structures of the cochlea and auditory nerve. Electrical current spread causes a broad region of the auditory nerve to be activated by stimulation at a single electrode and induces complex interactions between stimulation patterns from multiple electrodes (Shannon 1983; Townshend et al. 1987; McKay & McDermott 1996; Henry et al. 2000; McKay et al. 2001; Nelson & Donaldson 2001; Marozeau et al. 2015; Todd & Landsberger 2018; Goehring et al. 2020, 2021). Current spread also contributes to reducing the number of independent channels provided by the implant, as evidenced by speech perception for CI listeners not improving markedly when more than 4 to 8 independent channels are provided (Dorman et al. 1997; Hanekom & Shannon 1998; Friesen et al. 2001; Fu & Nogaki 2005), although more recent investigations suggest that improvements in speech perception can still be gained with more than 8 to 12 independent channels for some device types and electrode configurations (Croghan et al. 2017; Schvartz-Leyzac et al. 2017; Berg et al. 2019). Current spread can also impair the use of spectral and temporal cues, such as pulse-rate cues for perceptually segregating pulse trains applied to different electrode channels (Carlyon et al. 2007), inherent masker fluctuations for masking release of speech-in-noise (Oxenham & Kreft 2014), and differences in voice characteristics, such as fundamental frequency and timbre, for segregating competing talkers (Stickney et al. 2004). The firing pattern of the spiral ganglion neurons is also affected by temporal interactions (Boulet et al. 2016), and temporal charge interactions could further distort the stimulus envelope.

Some authors have reported that low stimulation rates, which increase the time interval between consecutive pulses and thus reduce the potential for pulse interactions, produced superior intelligibility and/or preference ratings compared to higher rates (Vandali et al. 2000; Balkany et al. 2007; Park et al. 2012; Brochier et al. 2017). However, in contrast, several other publications demonstrated benefits to intelligibility or preference at high stimulation rates (Fu & Shannon 2000; Kiefer et al. 2000; Loizou et al. 2000; Arora et al. 2011), or found no differences between outcomes for rates from 200 to 5000 pulses per second (Lawson et al. 1996; Friesen et al. 2005; Weber et al. 2007; Berg et al. 2023; Arora et al. 2024). Although reduced stimulation rates may reduce pulse interactions, they also provide reduced sampling of the speech envelope, which may lead to poor representation of speech features characterized by fast envelope fluctuations, such as consonant release bursts and vowel onsets.

Modifications to the speech processing strategy could provide an alternative method for sampling the temporal envelope, for instance, by providing sparser sampling of the envelope in the modulation troughs, given that CI listeners are insensitive to modulations in the lower half of their dynamic range (Monaghan et al. 2022). For example, the Fundamental Asynchronous Stimulus Timing (FAST) strategy greatly reduces the number of stimulation pulses by coding each envelope peak with a single electric pulse (Smith et al. 2013). Although initial investigations of the FAST strategy indicated that speech outcomes with FAST were not different from those obtained with a clinical strategy (Smith et al. 2013, 2014), a subsequent clinical trial with newly implanted CI recipients demonstrated worse speech intelligibility in speech-shaped and multi-talker noise with FAST than with a clinical strategy (National Library of Medicine, NCT02698787 2020) potentially due to under-sampling of the temporal envelope.

Rather than providing a single electric pulse at each envelope peak, the temporal integrator processing strategy (TIPS) uses a model of temporal masking to select a subset of electrical stimulation pulses from the stimulation pattern (Lamping et al. 2020), thereby reducing the potential for charge interactions while providing precise sampling at the envelope peaks. After using TIPS to remove half of the pulses in the stimulation pattern, Lamping et al. (2020) found that TIPS significantly improved the speech-reception threshold by 2.4 dB in the presence of speech-shaped noise relative to a CIS strategy. While this finding is promising, further evaluations of TIPS are required to replicate the effect of TIPS in speech-shaped noise with an additional set of listeners and to establish the effect of TIPS in conditions that more closely approximate everyday listening for CI recipients. In particular, TIPS must be evaluated in a background noise containing multiple competing talkers, which reflects a realistic and challenging listening condition for CI recipients (Cullington & Zeng 2008; Chen et al. 2023). TIPS must also be evaluated in conjunction with the Advanced Combination Encoder (ACE) strategy (McDermott et al. 1992; Vandali et al. 2000), as TIPS has been investigated with Cochlear devices (Sydney, Australia) and ACE is the strategy most commonly used by recipients of those devices. ACE selects N out of M (N-of-M) channels with the highest amplitude in each cycle for stimulation, thereby reducing the number of active electrodes and therefore the potential for pulse interactions in each stimulation cycle. This study extends the previous investigation of TIPS to (I) replicate the comparison to CIS in speech-shaped noise, (II) include a non-stationary multi-talker noise, and (III) compare to the ACE strategy, all in a new group of participants. In contrast to the investigation of Lamping et al. with Danish speakers and speech test materials, we evaluate a set of British English-speaking CI recipients using UK speech test materials. We also incorporate a high-frequency pre-emphasis filter in all testing conditions, to mimic the front-end processing that occurs in clinical Cochlear devices.

## MATERIALS AND METHODS

Two within-participant experiments measured speech perception outcomes with TIPS, compared to CIS (in experiment 1) and ACE (in experiment 2) reference strategies. All methods and statistical analyses were pre-registered on the Open Science Framework before data collection and statistical analyses (Shahidi et al. 2022).

### Participants

Twelve CI recipients participated in each experiment. Participant biographical data is provided in Table 1. A total of 16 CI recipients were recruited from the Emmeline Centre for CIs (Cambridge, England), and from the local population, with 8 individuals participating in both experiments 1 and 2 (Table 1). C52 was a bilateral CI user while all other participants were unilateral CI users. All testing was conducted unilaterally; if the participant had a hearing device contralateral to their tested implant, the contralateral hearing device was removed or turned off during testing. For experiment 1, the participants had a mean age of 60 years (range: 31 to 75 years), and 8 were male. For experiment 2, the participants had a mean age of 61 years (range: 31 to 75 years), and 4 were male. In both experiments, all participants were users of a Cochlear device and had at least 6 months of experience with their device. For all participants, the inter-phase gap and stimulation mode in their most frequently used clinical map were 8 µsec and MP1 + 2, respectively. Data collection required one session for each experiment, with sessions completed on different days. Data were collected at the Medical Research Council Cognition and Brain Sciences Unit in Cambridge, UK, in a testing room free from auditory and visual distractions, and ethical approval was obtained from the National Research Ethics Committee of the East of England before commencing the research. Each participant provided written informed consent before data collection and was paid for partaking in the study and reimbursed for any related travel expenses. For each experimental session, impedances were monitored before and after the session, and voltage requirements were always kept below the compliance limit for electrical stimulation.

**TABLE 1.** Information for each participant (indicated by identifier, ID) for experiments (Exp) 1 and 2

ID	Exp	Sex	Age at Testing (yrs)	Etiology	Duration of Deafness (yrs)	Implant Use (yrs)	Processor	Implant Type	Pulse Rate (pps)	Pulse Duration (µsec)	N Maxima
C27	1, 2	F	73	Unknown	Unknown	8.67	N7	CI512	900	25	10
C30	1	M	75	Measles	7	6.00	Kanso	CI512	900	25	8
C37	1, 2	F	70	Unknown	30	1.42	N7	CI632	900	25	10
C39	1, 2	M	67	Unknown	43	0.54	N7	CI622	900	37	8
C40	2	F	51	Unknown	36	0.83	N7	CI622	900	37	8
C41	2	F	75	Hereditary	27	3.25	N7	CI612	900	37	8
C42	2	F	62	Unknown	43	7.52	N8	CI522	900	62	7
C43	1, 2	F	67	Maternal rubella	53	1.33	N7	CI622	900	62	7
C44	1, 2	M	73	Unknown	42	5.00	N7	CI522	900	37	8
C45	1, 2	F	49	Unknown	4	0.85	N8	CI622	900	37	8
C50	1, 2	M	31	Hereditary	Unknown	8.05	N7	CI522	900	25	8
C51	1	M	70	Hereditary	31	0.97	N7	CI622	900	37	8
C52	1, 2	M	50	Physical trauma	33	9.29	N7	CI422	900	50	8
C53	2	F	69	Unknown	38	2.94	N7	CI622	900	37	8
C54	1	M	39	Meningitis	28	9.28	N7	CI422	900	50	8
C55	1	M	59	Hereditary	55	3.56	N7	CI622	500	37	8

Sex is indicated as female (F) or male (M). Duration of deafness indicates the years between profound hearing loss and receipt of a cochlear implant.

ID, identifier.

### Stimuli and Map Settings

The Nucleus MATLAB Toolbox (NMT, Swanson & Mauch 2006) emulation of CIS or ACE was used to transform acoustic signals into electric stimuli for experiments 1 and 2, respectively. All stimuli were first equalized to a fixed root mean square level of −20 dB FS relative to a 1 kHz pure tone calibrated to 65 dB SPL. Before CIS or ACE processing, the default pre-emphasis filter of the NMT was applied to the time-domain signal using a first-order Chebyshev Type II high-pass filter with a corner frequency of 4 kHz (and edge frequency of 8 kHz) and 3 dB of stopband attenuation. Other front-end processing algorithms that are available in clinically used devices, such as noise reduction and automatic gain control, were not applied. The base and saturation levels of the compression function applied by the NMT were set to 0 and 0.39, respectively. Electric stimuli were streamed to the recipient’s implant using the Nucleus Implant Communicator and the SP16 research processor provided by the Cochlear Corporation (Sydney, Australia). Stimulus presentation was controlled by custom MATLAB (The MathWorks, Inc., Natick, MA, USA) experimental research interfaces modified to interface with the Nucleus Implant Communicator.

For each participant, the reference map used the same settings, such as pulse rate, inter-phase gap, phase duration, and stimulation mode (i.e., ground electrode), as their most frequently used clinical map (Table 1). The electrodes specified by the reference map were active in the clinical map. During experiment 1, the CIS reference map was specified to have eight electrodes evenly spaced across the electrode array. The CIS map used eight electrodes (electrodes 6, 8, 10, 12, 14, 16, 18, and 20) for stimulus presentation for all participants except C43. The clinical map of participant C43 used a pulse duration of 62 µsec and a per-channel stimulation rate of 900 pulses per second, allowing only seven electrodes to be stimulated within one stimulation period. Therefore, participant C43’s experiment 1 maps used seven electrodes (electrodes 8, 10, 12, 14, 16, 18, and 20) for stimulus presentation. For experiment 2, all ACE reference maps used the same set of active electrodes and number of maxima as in the participants’ clinical map.

TIPS processing applies a model of temporal masking to identify and remove stimulation pulses that are unlikely to be perceived before stimulation at the electrode array. For each electrode channel, the output of the reference map is fed to a sliding temporal integrator (TI) (Oxenham & Moore 1994; Plack et al. 2002; McKay et al. 2013). The TI window, W(t), models forward and backward masking at time t relative to the center of the window (at time t=0):


$$\begin{array}{l} W\left(t\right)=\;\left\{ \begin{array}{l} \left(1-r\right)e^{\left(\frac{t}{T_{b1}}\right)}+re^{\left(\frac{t}{T_{b2}}\right)},\;\;\;\;\;\;t<0\;e^{\left(-\frac{t}{T_{a}}\right)},\;\;\;\;\;\;\;\;\;\;\;\;\;\;\;\;\;\;\;\;\;\;\;\;\;\;t\geq 0\; \end{array}\right. \end{array}$$

At time points before the center of the window, a double exponential function models the contributions of forward masking, using the time constants $T_{b1}$ and $T_{b2}$ and the weighting factor r. At time points following the center of the window, the contribution of backward masking is modeled by a backward exponential function using the time constant $T_{a}$. Here, we use the parameters $T_{b1}=4.6$ msec, $T_{b2}=16.6$ msec, $T_{a}=3.5$ msec, and r=0.17, as in Lamping et al. (2020).

**Fig. 1.**
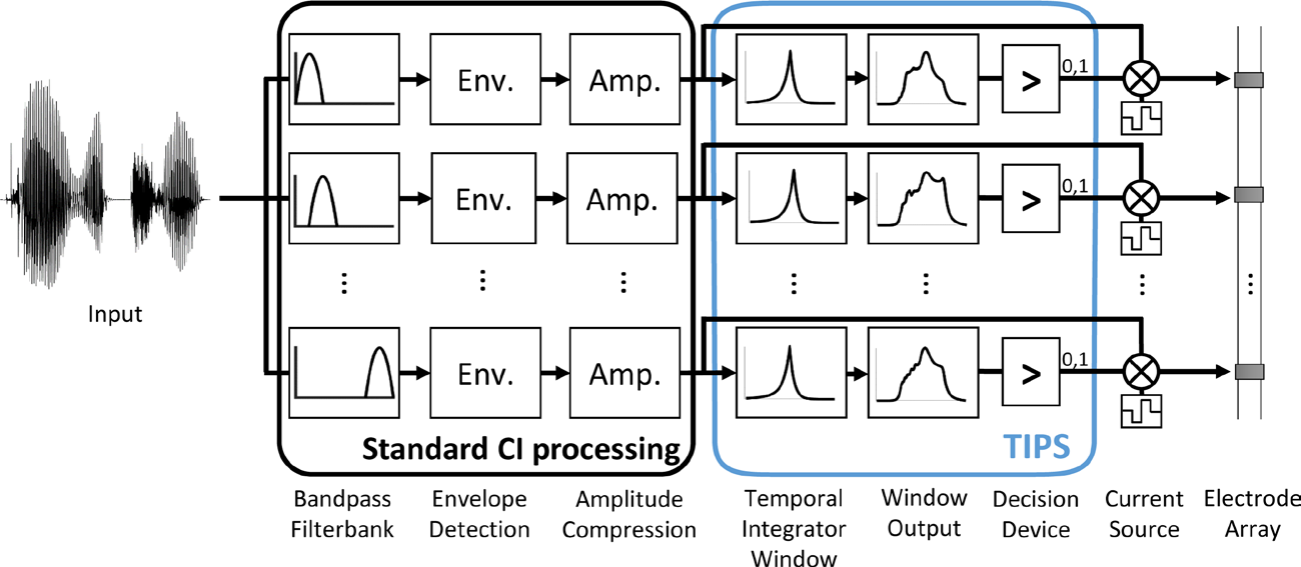
Block diagram of the TIPS, composed of the temporal integrator window and a decision device applied to the window output, implemented after the bandpass filterbank, Env. and Amp. steps in a standard CI processing strategy. Amp. indicates amplitude compression; CI, cochlear implant; Env., envelope extraction; TIPS, temporal integrator processing strategy.

To determine whether an electrical stimulation pulse should be removed, the TI window is applied via a convolution operation to the original stimulus and to a copy of the original stimulus in which the target pulse has been removed. In both cases, the TI window is centered on the location of the target pulse. The difference between the two window outputs is computed, and a decision criterion is applied to the log-transformed maximum of the difference. If the difference exceeds the criterion, the target pulse is maintained; otherwise, the target pulse is removed. Figure 1 illustrates the application of the TI window and the decision device to the output of envelope extraction-based strategies. In experiment 1, a decision criterion of 1.3 dB was used to remove 50% (TIPS50) of current pulses from the CIS baseline; this criterion was the same as in Lamping et al. (2020). In experiment 2, decision criteria of 1.3 and 1.62 dB were used to remove 33% (TIPS33) and 50% (TIPS50) of current pulses, respectively, from the ACE baseline after N-of-M selection. The first criterion was chosen to apply the same threshold as in experiment 1, and the second criterion was chosen to remove the same number of pulses as with TIPS applied to CIS in experiment 1.

### Loudness Scaling and Balancing

Before obtaining speech perception measurements, the most comfortable level (MCL) was obtained for each experimental strategy and balanced across the experimental strategies within each experiment. All loudness scaling and balancing procedures used broadband stimuli presented to the active electrodes in the experimental map, with the profile of T- and C-levels across electrodes determined by each participant’s clinical map.

First, the threshold level (T-level) from the clinical map was confirmed to be audible so that it could be used for the experimental strategy settings, as in Lamping et al. (2020). The profile of T-levels across electrodes from the clinical map in clinical current units (CUs, 1 CU = 0.157 dB) was used to set the relative level across electrodes and set to a minimum level, such that the lowest T-level in the profile was equal to one clinical current unit. The global current level was then gradually increased until the clinically set T-level was confirmed to be at an audible loudness level. T-levels were confirmed using unmodulated pulse trains of 400 millisecond duration, either presented to the subset of electrodes used by the CIS strategy in experiment 1 (as specified in Stimuli and map settings), or presented to N electrodes (with N equal to the number of maxima in each participant’s clinical map) in experiment 2. Using the confirmed T-levels, the MCL was then determined by gradually increasing the comfort level (C-level) of the experimental map to determine the most comfortable loudness level. The profile of C-levels from the clinical map was used to set the relative level across electrodes. To begin the MCL measurement procedure, the distance between the T- and C-level profiles was minimized. As the relative levels across electrodes likely differed across the T- and C-level profiles, as is common in clinical maps, the minimum level across all electrodes in the C-level profile was set to one clinical current unit above the maximum level across all electrodes in the T-level profile. For example, if the same electrode had both the highest level in the T-level profile and the lowest level in the C-level profile, this electrode would have a dynamic range of 1 CU at the start of the MCL measurement procedure, with the T- and C-levels for the remaining electrodes following the relative levels in the T- and C-level profiles, respectively. The C-level was then gradually increased while participants indicated the loudness level on a scale from 0 (“off”) to 10 (“too loud”). Once loudness level 7 (“loud but comfortable”) was reported, the C-level was reduced until loudness level 6 (MCL) was reported. The sentence, “Barry bought five large chairs,” from the British English matrix test (Hewitt 2008) was used to measure the C-level for all experimental strategies. The sentence was time-reversed to ensure the stimulus was unintelligible and not to provide speech-comprehension training for any experimental strategy.

Second, the experimental strategies were loudness-balanced across maps tested in the same experiment. Loudness balancing used a new time-reversed sentence, “Lucy bought nine green shoes,” from the British English matrix test as the stimulus. Two stimuli were presented, a standard stimulus and a signal stimulus, with each stimulus formed from the same sentence processed by a different experimental map, and the participant was asked to report which stimulus was louder. The signal stimulus was then adjusted by the experimenter by increasing or decreasing the global C-level of the stimulus as appropriate. This procedure was repeated until the two stimuli were reported as being equally loud. The standard and target stimuli were then switched, and the procedure was repeated, with the previously matched level used as the new C-level. This procedure was repeated for every pair of experimental strategies within each experiment until no discernible difference in loudness was reported across stimuli processed by all experimental strategies.

### Speech Perception Testing

After C-levels were balanced across strategies, speech perception testing was conducted in blocks, with each strategy tested in a single block. All speech material within each block was processed by the experimental strategy and used in the participants’ experimental settings determined during loudness scaling and balancing. The presentation order of strategies was counterbalanced across participants within each experiment, and both the participants and the study administrator were blinded to the conditions being tested. A break was provided before each block.

Within each block, participants were acclimatized to the experimental strategy before the speech perception assessments. First, 20 semantically complex sentences from the Clarity Enhancement Challenge (Barker et al. 2022) were presented, with a transcript provided for the first 10 sentences. Then, a 5-min excerpt from an audiobook of *Alice’s Adventures in Wonderland* by Lewis Carroll was presented with a transcript provided for the first 3 min of speech. Participants were instructed to read along with the provided transcripts as the speech was playing. The utterances selected for acclimatization differed across all testing blocks, but the order was held constant across blocks within each experiment, for example, block 1 in experiment 1 always used the same acclimatization speech material regardless of the experimental strategy being tested. All speech material throughout the experiments was spoken by a female speaker of Southern Standard British English.

Speech perception was then assessed as the speech quality rating in quiet and the speech intelligibility in noise. Speech quality was measured as a mean-opinion score (MOS) on a scale from 1 (“Bad”) to 5 (“Excellent”), and participants were instructed that a rating of 5 represented what they would like speech to sound like with their implant. After presentation of a single speech utterance from the British English Coordinate Response Measure test (Semeraro et al. 2017), participants entered their rating on an experimental interface, and the score assigned to four speech utterances was averaged to create one quality rating. Two speech quality ratings were measured in each block, immediately before (pre-test) and after (post-test) the speech-in-noise assessment.

Speech intelligibility in noise was measured as the speech-reception threshold (SRT), defined as the signal to noise ratio (SNR) at which 50% of words are correctly reported. SRTs were measured adaptively using the British English matrix test (Hewitt 2008), a closed-set matrix test in which each sentence is composed of a name, verb, number, adjective, and noun, with 10 possible options for each word. There are 200 recorded sentences in the British English matrix test, divided into 20 lists with 10 sentences per list. During testing, participants entered their responses by selecting the corresponding words on an experimental interface, and each response was automatically scored. For procedural learning, 20 sentences (from two lists) were presented without background noise before SRT measurement. For each adaptive SRT measurement, two lists were used, and the SRT was estimated as the average SNR at the last eight reversals. Within each adaptive track, the SNR was initially set to 0 dB, and the next stimulus was generated at an SNR targeting 50% speech recognition by varying the level of the noise, with the next SNR determined using the adaptive method of Brand and Kollmeier (2002). Each stimulus used a randomly sampled segment of noise, and the SNR was bounded between -15 and +30 dB. The final SRT measurement was obtained by averaging the SRT estimated over two adaptive tracks. If fewer than eight reversals were achieved within a single adaptive track or the SRT between the two measurements differed by more than 4 dB, an additional SRT measurement was performed, and the average of the three measurements was taken as the final SRT measure.

The SRT was measured in a speech-shaped noise (SSN) and in a multi-talker babble noise in experiment 1, and only in a multi-talker babble noise in experiment 2. The SSN had the same long-term average spectrum as the British English matrix sentences. The multi-talker babble noise was comprised of a mixture of competing speech from 20 English talkers (8 male, 12 female) and was recorded at Auditec, Inc. (St. Louis, MO, USA). The order of noises was counterbalanced across participants and strategies in experiment 1.

### Power Savings Estimation

TIPS impacts overall power consumption by reducing the number of stimulation pulses and by increasing the stimulation level per pulse. The power sent to the radio frequency (RF) coil of a CI is used to stimulate the electrodes and keep the implant electronics running. We estimated that the power sent to the RF coil constitutes 90% of the total power required by the implant and that this component of power consumption drops linearly with the stimulation level, as described in Lamping et al. (2020). Net power savings due to TIPS were thus estimated as linear changes to the 90% of power allocated to the RF coil, first accounting for the reduction in the number of stimulation pulses due to TIPS and then for the increase in charge due to an increase in the global C-level used for stimulation. This method for estimating the net power saving is presented in equation form in Supplemental Digital Content 1, https://links.lww.com/EANDH/B755.

### Statistical Analysis

Pre-registered (Shahidi et al. 2022) statistical analyses were performed using linear mixed-effects modeling (LMM), with a separate model for each outcome measure (SRT and quality rating) and experimental TIPS map. For speech intelligibility in noise, SRT outcomes were modeled with fixed effects for the application of TIPS (categorical variable with reference category: baseline strategy) and a random effect of participant. For SRT outcomes in experiment 1, an additional fixed effect was included for the noise type (categorical variable with reference category: SSN), and a term was included to model the interaction between noise type and TIPS application. For the speech quality assessments, ratings were modeled with fixed effects for the application of TIPS (categorical variable with reference category: baseline strategy) and the order of the assessment (categorical variable with reference category: Pre-test measurement). LMMs were implemented using the *lme4* package in R (Bates et al. 2015). *p* Values for the fixed effects terms were calculated from *F* tests (using Satterthwaite approximation of denominator degrees of freedom) and for the random effect using likelihood ratio tests (Kuznetsova et al. 2015). Post hoc comparisons were performed using contrasts of estimated least-square marginal means using the *emmeans* package in R (Searle et al. 1980; Lenth 2018) and the *lme4* model object. *p* Values were corrected for multiple comparisons using the Tukey method, and significant differences are reported using an α (type I error) level of 0.05. To determine 95% confidence intervals (95% CI) for the effect of TIPS on mean outcome measures for each participant, the SE of the difference between sample means across strategies was first computed and then multiplied by a *t* score (informed by the degrees of freedom of the SE estimate). All mean differences with CIs that do not overlap with zero are reported in the results section.

We used a sequential analysis design in which an interim analysis of the effect of strategy on SRT outcomes was conducted after testing was completed for 8 participants in either experiment. A second final analysis was then performed after the testing was completed for 12 participants. Type I error (α) was distributed over the two planned analyses using the adjustment method of O’Brien and Fleming (1979), yielding nominal α levels for significant differences of 0.0184 and 0.0440 at the first and second planned analyses, respectively. This method was selected to stringently control for false positives, but not false negatives, at the interim analysis to ensure significant effects observed at the interim analyses were robust.

## RESULTS

### Loudness Balancing and Estimated Power Savings

The increase in the charge of the global C-level needed to achieve the same comfortable listening level as that achieved with the baseline strategy is presented in Table 1, both in nanoCoulomb (nC) and as a percentage increase. The estimated net power savings, accounting for the increase in charge and the reduction in the number of stimulation pulses, are also presented in Table 2. In comparison to the CIS baseline in experiment 1, TIPS50 required the charge to be increased by approximately 6.05% (1.22 nC) on average across the 12 participants, leading to an average net power saving of 42.3%. In comparison to the ACE baseline in experiment 2, TIPS33 required the charge to be increased by approximately 13.14% (1.48 nC), leading to an average net power saving of 21.8% and TIPS50 required the charge to be increased by approximately 25.45% (3.08 nC), leading to an average net power saving of 33.6%.

**TABLE 2.** The charge difference in the global C-level necessary to achieve the same loudness level for the TIPS conditions relative to the CIS reference (in experiment 1) or the ACE reference (in experiment 2), in nC and as a percentage (%), and the estimated net power savings conferred by TIPS relative to the respective reference as a percentage (%), assuming the power sent to the coil constitutes 90% of the total power required by the device

ID	Experiment 1: CIS + TIPS 50%			Experiment 2: ACE + TIPS 33%			Experiment 2: ACE + TIPS 50%		
	Charge (nC)	Charge (%)	Power (%)	Charge (nC)	Charge (%)	Power (%)	Charge (nC)	Charge (%)	Power (%)
C27	0.00	0.00	45.00	1.14	5.57	26.66	2.35	11.44	39.85
C30	0.00	0.00	45.00	–	–	–	–	–	–
C37	0.47	1.82	44.18	1.20	5.57	26.66	1.61	7.49	41.63
C39	1.54	11.44	39.85	2.60	28.77	12.74	3.93	43.50	25.42
C40	–	–	–	1.62	11.44	23.13	2.80	19.79	36.09
C41	–	–	–	0.53	5.57	26.66	2.10	21.98	35.11
C42	–	–	–	2.11	15.54	20.67	6.99	51.49	21.83
C43	3.15	15.54	38.01	1.21	5.57	26.66	2.05	9.45	40.75
C44	0.00	0.00	45.00	1.05	7.49	25.50	1.61	11.44	39.85
C45	1.89	13.48	38.94	3.25	33.50	9.89	4.47	46.12	24.25
C50	1.43	5.57	42.49	1.85	13.48	21.91	3.64	26.46	33.09
C51	0.59	3.68	43.34	–	–	–	–	–	–
C52	0.56	1.82	44.18	−0.90	−3.55	32.13	1.89	7.49	41.63
C53	–	–	–	2.06	28.77	12.74	3.49	48.78	23.05
C54	1.03	3.68	43.34	–	–	–	–	–	–
C55	3.98	15.54	38.01	–	–	–	–	–	–
Avg	1.22	6.05	42.28	1.48	13.14	21.78	3.08	25.45	33.55

ACE, advanced combination encoder; CIS, continuous interleaved sampling; ID, identifier; nC, nanoCoulomb; TIPS, temporal integrator processing strategy.

### Speech Perception: TIPS Versus CIS

**Fig. 2.**
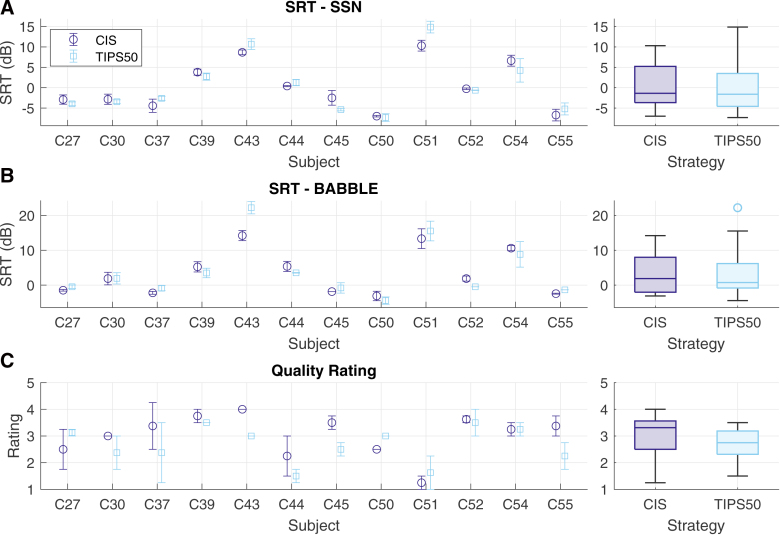
Speech perception outcomes from experiment 1 for stimuli processed by CIS (purple) and TIPS50 (blue), measured as (A) the SRT in SSN, (B) the SRT in BABBLE, and (C) the mean-opinion score quality rating. Individual results (left panels) are presented as the mean scores, with error bars indicating the SE of the mean, and group results (right panels) are presented as boxplots indicating the median, inter-quartile range, and non-outlier maximum and minimum. BABBLE indicates multi-talker babble noise; CIS, continuous interleaved sampling; SRT, speech-reception threshold; SSN, speech-shaped noise.

Figure 2 presents the speech perception outcomes obtained in experiment 1. SRTs in dB SNR obtained with the CIS and TIPS50 strategies are presented for SSN (Fig. 2A) and multi-talker noise (Fig. 2B) as individual (left panels) and group (right panels) results. In SSN, participants obtained a range of SRTs from −7.33 dB (for C50 with TIPS50) to 14.87 dB (for C51 with TIPS50). Group-level results indicate similar SRT outcomes with CIS and TIPS50, with median SRTs of −1.38 dB with CIS and −1.62 dB with TIPS50. Although performance appeared to vary across participants, estimates for the difference between sample means computed for each participant did not indicate differences in SRT outcomes between CIS and TIPS50.

In the multi-talker noise, SRTs ranged from −4.43 (for C50 with TIPS50) to 22.21 dB (for C43 with TIPS50). Median group SRTs were 1.88 dB with CIS and 0.74 dB with TIPS50. Performance varied across participants, with TIPS50 having no consistent effect on the SRT for most participants and worsening the SRT for others (e.g., by raising the SRT by 1.09 dB [95% CI: 0.51 to 1.67 dB] for C55). Generally, participants’ performance with TIPS relative to their performance with CIS appeared to correlate across the two noise conditions, but this failed to reach significance in a post hoc Spearman rank correlation analysis (*r*_s_ = 0.552; *p* = 0.067). Given the small sample size, this post hoc analysis was underpowered, achieving only 69% power. To achieve 90% power, a sample size of 22 participants would be required.

LMM indicated no significant main effect of processing strategy on SRT outcomes [*F*(1,36) = 0.389; *p* = 0.537] when comparing against the nominal *p* value of 0.0440 established in the sequential analysis. There was a significant main effect of noise type [*F*(1,36) = 42.174; *p* < 0.0001] but no interaction between strategy and noise type [*F*(1,36) = 0.087; *p* = 0.770]. A post hoc comparison for the main effect of noise type showed that performance was significantly worse in the multi-talker noise than in SSN [*t*(39.3) = −6.218; *p* < 0.0001], with the multi-talker noise resulting in an estimated 3.66 dB increase in the SRT. For all LMMs presented in this work, tables detailing the model formulae, coefficients, upper and lower CIs, and the relevant contrasts are included in Supplemental Digital Content 2, https://links.lww.com/EANDH/B756.

Figure 2C presents the MOS quality ratings for the CIS and TIPS50 strategies. Although some participants assigned nominally higher ratings to TIPS than to CIS (such as C50 [0.50 point increase, 95% CI: 0.50 to 0.50]), other participants assigned lower ratings to TIPS than to CIS (such as C43 [1.00 point decrease, 95% CI: 1.00 to 1.00]). Group-level results indicate that TIPS earned overall lower quality ratings than CIS, with median quality ratings of 3.31 for CIS and 2.75 for TIPS50.

The LMM indicated a main effect of strategy [*F*(1, 36) = 4.744; *p* = 0.036] and a main effect of the order of quality assessment (either pre- or post-SRT measurement) [*F*(1, 36) = 7.160; *p* = 0.011] but no interaction effect between strategy and order [*F*(1,36) = 0.190; *p* = 0.666]. Post hoc comparisons indicated that TIPS50 earned lower quality ratings than CIS [*t*(39.3) = 2.085; *p* = 0.044] by an estimated 0.365 points on the 1 to 5 rating scale and that, averaged across strategies, ratings were generally 0.448 points larger when taken after SRT measurement compared to before [*t*(39.3) = −2.562; *p* = 0.014].

### Speech Perception: TIPS Versus ACE

**Fig. 3.**
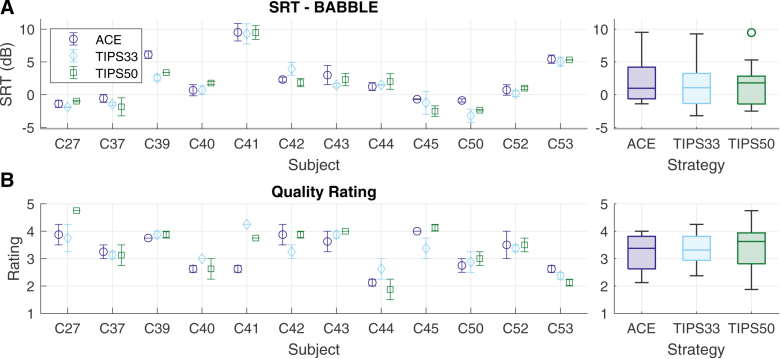
Speech perception outcomes from experiment 2 for stimuli processed by ACE (purple), TIPS33 (blue), and TIPS50 (green), measured as (A) the SRT in multi-talker babble noise (BABBLE) and (B) the mean-opinion score quality rating. Individual results (left panels) are presented as the mean scores, with error bars indicating the SE of the mean, and group results (right panels) are presented as boxplots indicating the median, inter-quartile range, and non-outlier maximum and minimum, with outliers indicated by circular markers. ACE, advanced combination encoder; SRT, speech-reception threshold.

Figure 3 presents the speech perception outcomes obtained with ACE, TIPS33, and TIPS50 in experiment 2. SRTs in dB SNR obtained in multi-talker noise are presented in Figure 3A. In multi-talker noise, participants obtained a range of SRTs from −3.21 dB (for C50 with TIPS33) to 9.52 dB (for C41 with ACE). The group-level results indicate median SRTs of 0.97 dB with ACE, 1.07 dB with TIPS33, and 1.81 with TIPS50. Participants again demonstrated different trends in speech perception across strategies. Notably, the SRT was improved for some participants by TIPS33 (e.g., by lowering the SRT by 3.55 dB [95% CI: 0.38 to 6.72 dB] for C39) and/or by TIPS50 (e.g., by lowering the SRT by 2.76 dB [95% CI: 0.32 to 5.19 dB] for C39 and by 1.49 dB [95% CI: 0.31 to 2.66 dB] for C50). No participant in experiment 2 demonstrated worsened SRT scores with either TIPS condition. Linear mixed-effects modeling, with separate models for each of the TIPS strategies, revealed no main effect of strategy for TIPS33 [(1,12) = 3.961; *p* = 0.070] or TIPS50 [*F*(1,12) = 2.589; *p* = 0.134].

Figure 3B presents the MOS quality ratings for the ACE, TIPS33, and TIPS50 strategies. Participants demonstrated individual trends in quality ratings across strategies, with one participant consistently rating TIPS as having higher quality than ACE, (C41 for TIPS33 [1.63 point increase, 95% CI: 1.09 to 2.16] and TIPS50 [1.13 point increase, 95% CI: 0.59 to 1.66]). The LMM indicated no significant effect of strategy on quality ratings for TIPS33 [*F*(1,36) = 0.548; *p* = 0.464] or TIPS50 [*F*(1,36) = 2.524; *p* = 0.121], no significant effect of rating order on quality ratings for TIPS33 [*F*(1,36) = 0.548; *p* = 0.464] or TIPS50 [*F*(1,36) = 2.524; *p* = 0.121], and no interaction between strategy and rating order for TIPS33 [*F*(1,36) = 0.548; *p* = 0.464] or TIPS50 [*F*(1,36) = 0.158; *p* = 0.694].

## DISCUSSION

The effect of TIPS processing on speech intelligibility in noise and speech quality was evaluated against a CIS reference strategy in experiment 1 and an ACE reference strategy in experiment 2, extending the evaluation of Lamping et al. (2020) to include conditions that more closely reflect everyday listening for CI recipients.

### Speech Outcomes and Participant Variability

Speech perception in noise was similar for TIPS compared to the CIS baseline in experiment 1 and the ACE baseline in experiment 2. The SRT measurements are reliable, given the highly significant (*p* < 0.0001) increase in the SRT in multi-talker babble compared to SSN in experiment 1, in line with previous reports (Oba et al. 2011; Taitelbaum-Swead & Fostick 2022), and high intra-class correlations between the two SRT measurements of 0.87 in experiment 1 and 0.89 in experiment 2. SRTs measured with CI recipients using an adaptive matrix speech test have reported test-retest precision ranging from ±0.3 to ±1.3 dB in a test-matched SSN (Hey et al. 2014; Theelen-van Den Hoek et al. 2014; Dietz et al. 2015; Potts et al. 2023) and ±1.2 dB in a four-talker babble noise (Potts et al. 2023). While these measures give an idea of the precision of the matrix test with CI recipients, it should be noted that test precision has not been reported with CI recipients for the British English matrix test. In experiment 2, some participants demonstrated improvements in the SRT with TIPS outside of the reported measurement precision of the matrix test when tested against the participants’ ACE strategy, most notably C39 (3.55 dB improvement with TIPS33 and 2.76 dB improvement with TIPS50) and C50 (1.49 dB improvement with TIPS50). Increases in (worsening of) the SRT in excess of the reported matrix test precision were not observed for any participant in any condition.

As noted by Lamping et al. (2020), it is possible that some listeners will experience greater changes in speech-in-noise perception with TIPS than others. To investigate this hypothesis, a post hoc analysis was performed using a univariate factorial analysis of variance with factors for participant, strategy, and an interaction between participant and strategy, and applied to individual SRT measurements. The exploratory analysis revealed no significant interaction of participant and strategy in experiment 1 in SSN [*F*(11) = 1.184; *p* = 0.336] or babble [*F*(11) = 1.145; *p* = 0.368], or in experiment 2 with TIPS33 [*F*(11) = 1.264; *p* = 0.300] or TIPS50 [*F*(11) = 1.152; *p* = 0.368]. Overall, the exploratory analysis did not support the idea that trends in speech-in-noise perception across strategies varied with the participant, suggesting that the variability in outcomes observed across participants does not reflect individual participants’ predisposition to changes in speech intelligibility with TIPS.

Most listeners reported difficulty in understanding speech with the baseline CIS strategy, which differed from their clinical everyday strategy mainly in the use of a restricted subset of each participant’s active electrode channels. Difficulty with the CIS strategy is reflected in the higher SRTs in babble obtained in experiment 1 with CIS (median: 1.88 dB) than in experiment 2 with ACE (median: 0.97 dB) and may have made the addition of TIPS processing harder to adapt to, given that only limited acclimatization was provided. Furthermore, if listeners could not obtain above-chance performance on the matrix test in quiet with the CIS baseline strategy, they were deemed ineligible for experiment 1. This inclusion criterion, which excluded four participants from experiment 1, may have biased data collection toward selecting listeners who performed well with the CIS baseline. Speech quality ratings tended to be lower for TIPS when compared to the CIS baseline in experiment 1 [*t*(39.3) = 2.085; *p* = 0.044], but were not significantly different for TIPS when compared to the ACE baseline in experiment 2. Difficulty with the CIS strategy may have contributed to lower quality ratings assigned to TIPS, given the aforementioned reasons. Interestingly, most participants in experiment 2 (10 out of 12) indicated that at least one of the TIPS maps resulted in speech of nominally equivalent or better quality than that produced by the ACE strategy, despite ACE being the participants’ everyday clinical program. Although quality ratings in experiment 2 tended to be higher for the TIPS strategies than the ACE strategy, only one participant (C41) consistently rated TIPS-processed stimuli as higher quality than ACE-processed stimuli, with CIs for the change in quality rating with TIPS not encompassing zero.

It is possible that participants who performed better with TIPS in the speech-in-noise task assigned TIPS higher speech quality ratings, and likewise for the baseline strategy. To investigate whether the difference in quality ratings was related to the difference in speech-in-noise perception, Spearman rank correlation was used to assess the relationship between the change in the SRT and the change in the quality rating with TIPS from the baseline strategy. This *post hoc* analysis indicated no significant relationship in experiment 1 for TIPS50 in SSN (*r*_s_ = 0.183; *p* = 0.569) or in multi-talker babble noise (*r*_s_ = −0.014; *p* = 0.965) and no significant relationship in experiment 2 for TIPS33 (*r*_s_ = 0.018; *p* = 0.957) or for TIPS50 (*r*_s_ = −0.180; *p* = 0.576) in multi-talker babble noise. Hence we found no evidence that participants who rated speech processed by TIPS to be of better quality than the baseline strategy tended to show a benefit in the speech-in-noise task (and vice versa), consistent with the idea that the SRT and speech quality tasks measure distinct aspects of speech perception.

Although this study did not find evidence that TIPS improves speech perception at the group level, it appears that TIPS may allow for the removal of a considerable proportion of pulses from the stimulation pattern without any detriment to speech perception, even in challenging listening scenarios containing multiple competing talkers.

### Power Savings

The power required for stimulation by the CI could be considerably reduced by using the TI window to guide pulse removal. The estimated net power saving of 42.3% for TIPS50 applied to the CIS strategy is in line with that reported by Lamping et al. (2020), in which TIPS50 led to an approximate average net power saving of 40.95% over the CIS baseline. This outcome suggests that, on average, TIPS50 required the same increase in the global C-level across participants in the Lamping et al. study and the current study. The approximate average net power saving conferred by TIPS50 when applied after ACE processing (33.6%) was lower than that obtained with CIS in experiment 1, due to greater increases in C-level required to maintain a comfortable listening level when TIPS processing was applied to ACE. This result suggests less charge summation occurred with ACE than with CIS due to the greater spatial distribution of stimulation pulses across the electrode array, and reflects an estimation of power savings under processing conditions that are more likely to reflect the everyday processing strategy of a Cochlear device recipient.

### Comparison to Prior TIPS Evaluation

There are notable differences in the speech perception outcomes observed with TIPS when comparing the current study to the earlier investigation of TIPS presented in Lamping et al. (2020). In particular, the 2.4 dB SRT benefit of TIPS50 in SSN observed by Lamping et al. was not replicated for the listeners tested in the current study, with TIPS resulting in no difference to the SRT on a group level. An exploratory analysis was conducted to determine whether the SRTs obtained in SSN in experiment 1 differed from those observed in Lamping et al. by fitting an LMM to the combined dataset with model fixed effects for strategy, experiment, and an interaction of strategy and experiment, and a random effect of participant. Bonferroni corrections were applied for multiple comparisons. The model revealed no effect of strategy [*F*(1,20) = 5.562; *p* = 0.057] or experiment [*F*(1,20) = 0.944; *p* = 0.686] on SRT measures. It is important to note that the model indicated a significant interaction of strategy and experiment [*F*(1,20) = 7.331; *p* = 0.027], likely due to differences in participants, strategy, or stimuli.

Differences in SRT outcomes with TIPS-processed stimuli across the two studies may have arisen from the inclusion of the pre-emphasis filter, the use of the British English matrix speech material, or the recruitment of different listeners in this study. The pre-emphasis filter, included in all clinically available Cochlear devices, increases the overall amplitude of pulses in high-frequency electrode channels, modifying the gain in these electrode channels. Without pre-emphasis, stimulation pulses in low-frequency electrode channels will likely have higher amplitudes than pulses in high-frequency electrode channels, and are therefore more likely to be selected for stimulation during N-of-M selection. Although the previous evaluation of TIPS did not include the pre-emphasis filter, the change in gain due to pre-emphasis was unlikely to produce changes in the pulse-removal behavior of TIPS. Not only are the relative amplitudes of pulses within each electrode channel unaffected by the pre-emphasis filter, but a preliminary analysis found no change in the proportion of pulses removed by TIPS with and without the pre-emphasis filter across criteria. On the other hand, the use of the British English matrix test, compared to the Danish matrix test used by Lamping et al. (2020), may have contributed to changes in pulse removal by TIPS as difference in articulation, such as those arising from differences in speaking rate, could change the relative modulation rate across channels and thus the pulses removed by TIPS. Lastly, the participants included in this assessment of TIPS may have played a large role in the measured effect of TIPS, given the large variability in outcomes generally observed across CI recipients. Lamping et al. found that listeners who obtained higher SRTs with CIS were more likely to benefit from TIPS50, and the SRTs observed in this study were generally lower than those observed by Lamping et al.. In addition, most listeners in this study obtained similar SRTs across strategies, unlike the listeners tested in Lamping et al., where larger differences were found across strategies for individual listeners. Given the diverse array of hearing outcomes generally observed across CI recipients, the effects that could be detected with the small sample size used in the current study would need to be large and consistently observed across participants. Future studies of TIPS should incorporate a larger sample size to provide robust evidence for TIPS efficacy and to facilitate the detection of more moderately sized effects. Such studies should also include listening conditions where masked-pulse removal may provide material benefits to perception, for instance, in the identification and discrimination of the fundamental frequency of modulated tones with implications for stream segregation and speech-in-noise perception. Overall, these results further emphasize the importance of strictly controlled experimental designs and experimental replication, as exemplified by previous investigations of speech processing strategies (e.g., MP3000 and FAST) which initially demonstrated speech perception advantages (Nogueira et al. 2005; Smith et al. 2013) that ultimately were not replicated (National Library of Medicine, NCT02698787 2020; Buechner et al. 2011).

### Real-World Potential

Despite the lack of significant improvements in SRT due to TIPS in the present study, the substantial power savings conferred by TIPS could still benefit CI recipients by lengthening battery life, reducing the size of the external battery, and/or providing power for computationally expensive front-end noise reduction algorithms. Further research is needed to refine the detection of masked pulses by the TIPS algorithm, for instance, by incorporating information from across electrode channels into the masking model. Such a refinement could further improve the efficacy of information transmission by the CI and thus the power savings conferred by TIPS. Whether similar speech or power savings benefits could be achieved through a simpler method for reducing the number of stimulation pulses, such as by lowering the stimulation rate, must also be determined.

It is also possible that speech perception benefits with TIPS may arise after longer acclimatization, such as after continuous use during a clinical take-home study. Take-home studies of experimental stimulation strategies have demonstrated improvements in speech-in-noise perception with extended use (Punte et al. 2014; van Groesen et al. 2023), although these improvements might arise either from “genuine” perceptual learning or from other factors such as increased familiarity with the testing materials, as discussed elsewhere (Carlyon & Goehring 2021). Such a take-home study would additionally facilitate the evaluation of TIPS in a diverse array of real-world listening scenarios and the measurement of the power savings conferred by TIPS when used daily on a clinical device.

For TIPS to be implemented on clinical devices, the TIPS algorithm must be amended for real-time processing. To estimate the contribution of backward masking to a stimulation pulse, the TI window acts on a temporal segment of the stimulation pattern that extends 75 msec beyond the stimulation pulse in question. If implemented in a clinical device, this backward masking window would require stimulation to be delayed in order to collect sufficient temporal frames for TIPS to act upon. Such a delay may be perceivable to CI recipients as audio-visual asynchrony or a delay in own-voice perception (Hay-McCutcheon et al. 2009; Goehring et al. 2018), potentially harming speech perception and production. A real-time TIPS implementation would require adapting the TI window to use only information from the current and past stimulation frames, enabling TIPS-processed stimuli to be constructed and delivered with no discernible delay.

## SUMMARY AND CONCLUSIONS

TIPS removes pulses from the CI stimulation pattern using a model of temporal masking. Speech perception in noise and speech quality ratings with TIPS were assessed in conjunction with the CIS and ACE processing strategies in two experiments, each testing 12 CI recipients. The experiments suggest that TIPS could result in substantial power savings of up to 42%, without compromising speech quality or speech intelligibility in noise. Although there were some individual differences, the improvement in the average SRT previously found by Lamping et al. (2020) was not observed in the group-level SRTs in experiments 1 and 2. Despite the lack of consistent improvements in SRT due to TIPS, the power savings conferred by TIPS could benefit CI recipients by increasing battery life, improving device form factor, and providing power for front-end noise reduction algorithms.

## ACKNOWLEDGMENTS

The authors thank the study participants for their time and effort.

## Supplementary Material

## OPEN PRACTICES

This manuscript qualifies for an Open Data badge, Open Materials badge, and Preregistered badge. The materials and data have been made publically available at https://osf.io/fqa5j/ and https://osf.io/7rftv. More information about the Open Practices Badges can be found at https://journals.lww.com/ear-hearing/pages/default.aspx.
