# Supplementary material for: An Evaluation of the Temporal Integrator Processing Strategy for Cochlear Implants in Comparison to the Clinical Strategy and in Multi-Talker Noise
Source: Ear Hear. 2025 Nov 20;47(2):453–64. doi: 10.1097/AUD.0000000000001741 (PMC12904243; doi:10.1097/AUD.0000000000001741)
Supplement: Supplementary file 1 [file aud-47-453-s001.pdf]

Supplemental Digital Content 1

Considering the proportion increase in charge needed to reach a comfortable listening level, δ_{charge}, and the proportion of pulses removed by TIPS, δ_{pulses}, net power savings with TIPS are estimated as:

$$Net\ Power\ Savings = 1 - \left(0.1 + 0.9 \times \left(1 + \delta_{charge}\right) \times \left(1 - \delta_{pulses}\right)\right)$$

$$= 0.9 \times (1 - (1 + \delta_{charge}) \times (1 - \delta_{pulses}))$$
