# Supplementary material for: An Evaluation of the Temporal Integrator Processing Strategy for Cochlear Implants in Comparison to the Clinical Strategy and in Multi-Talker Noise
Source: Ear Hear. 2025 Nov 20;47(2):453–64. doi: 10.1097/AUD.0000000000001741 (PMC12904243; doi:10.1097/AUD.0000000000001741)
Supplement: Supplementary file 2 [file aud-47-453-s002.pdf]

Supplemental Digital Content 2

Experiment 1: CIS vs. TIPS50

Table S1. Linear mixed effects modelling for effect of strategy (Continuous Interleaved Sampling, CIS, or Temporal Integrator Processing Strategy, TIPS50) and noise (speech-shaped noise or multi-talker noise) on speech reception threshold (SRT).

Model: SRT ~ Strategy + Noise + Strategy:Noise + (1 | Participant)

Fixed Effect	Coefficient	Lower CI	Upper CI	Num DF	Den DF	F	p
Strategy	0.1693	-1.2584	1.5970	1	36	0.3890	0.5367
Noise	3.1930	**1.7653**	**4.6207**	1	36	42.1736	**1.527e-07**
Strategy:Noise	0.3040	-1.7151	2.3231	1	36	0.0871	0.7696

CI – confidence interval; DF – degrees of freedom; Num – numerator; Den - denominator

Table S2. Estimated marginal means for the contrast strategy (Continuous Interleaved Sampling, CIS, and Temporal Integrator Processing Strategy, TIPS50) and noise type (speech-shaped noise, SSN, and multi-talker noise, MTN) using the linear mixed effects model presented in Table S1.

Contrast	Estimate	SE	DF	***t***	p
CIS-TIPS50	-0.321	0.538	39.3	-0.597	0.5538
SSN-MTN	-3.34	0.538	39.3	-6.218	**<0.0001**

SE – standard error; DF – degrees of freedom

Table S3. Linear mixed effects modelling for effect of strategy (Continuous Interleaved Sampling, CIS, or Temporal Integrator Processing Strategy, TIPS50) and order (either prior to or after speech-in-noise testing) on mean opinion score quality ratings.

Model: Rating ~ Strategy + Order + Strategy:Order + (1 | Participant)

Fixed Effect	Coefficient	Lower CI	Upper CI	Num DF	Den DF	F	p
Strategy	-0.2917	-0.7557	0.1723	1	36	4.7435	**0.03604**
Order	0.5208	**0.0568**	**0.9848**	1	36	7.1597	**0.01115**
Strategy:Order	-0.1458	-0.8020	0.5104	1	36	0.1897	0.66573

CI – confidence interval; DF – degrees of freedom; Num – numerator; Den - denominator

Table S4. Estimated marginal means for the contrast strategy (Continuous Interleaved Sampling, CIS, and Temporal Integrator Processing Strategy, TIPS50) and order (either prior to, PreTest, or after, PostTest, speech-in-noise testing) using the linear mixed effects model presented in Table S3.

Contrast	Estimate	SE	DF	***t***	p
CIS-TIPS50	0.365	0.175	39.3	2.085	**0.0436**
PreTest-PostTest	-0.448	0.175	39.3	-2.562	**0.0144**

SE – standard error; DF – degrees of freedom

Supplemental Digital Content 2

<u>Experiment 2: ACE vs TIPS33</u>

Table S5. Linear mixed effects modelling for effect of strategy (Advanced Combination Encoder, ACE, or Temporal Integrator Processing Strategy, TIPS33) on speech reception threshold (SRT).

Model: SRT ~ Strategy + (1 | Participant)

Fixed Effect	Coefficient	Lower CI	Upper CI	Num DF	Den DF	F	p
Strategy	-0.7191	-1.4273	-0.0109	1	12	3.9607	0.06985

CI – confidence interval; DF – degrees of freedom; Num – numerator; Den - denominator

Table S6. Linear mixed effects modelling for effect of strategy (Advanced Combination Encoder, ACE, or Temporal Integrator Processing Strategy, TIPS33) and order (either prior to or after speech-in-noise testing) on mean opinion score quality ratings.

Model: Rating ~ Strategy + Order + Strategy:Order + (1 | Participant)

Fixed Effect	Coefficient	Lower CI	Upper CI	Num DF	Den DF	F	p
Strategy	0.1875	-0.1635	0.5385	1	36	0.5480	0.4639
Order	0.2292	-0.1219	0.5802	1	36	1.1434	0.2921
Strategy:Order	-0.1875	-0.6839	0.3089	1	36	0.5480	0.4639

CI – confidence interval; DF – degrees of freedom; Num – numerator; Den - denominator

<u>Experiment 2: ACE vs TIPS50</u>

Table S7. Linear mixed effects modelling for effect of strategy (Advanced Combination Encoder, ACE, or Temporal Integrator Processing Strategy, TIPS50) on speech reception threshold (SRT).

Model: SRT ~ Strategy + (1 | Participant)

Fixed Effect	Coefficient	Lower CI	Upper CI	Num DF	Den DF	F	p
Strategy	-0.5096	-1.1304	0.1112	1	12	2.5886	0.1336

CI – confidence interval; DF – degrees of freedom; Num – numerator; Den - denominator

Table S8. Linear mixed effects modelling for effect of strategy (Advanced Combination Encoder, ACE, or Temporal Integrator Processing Strategy, TIPS50) and order (either prior to or after speech-in-noise testing) on mean opinion score quality ratings.

Model: Rating ~ Strategy + Order + Strategy:Order + (1 | Participant)

Fixed Effect	Coefficient	Lower CI	Upper CI	Num DF	Den DF	F	p
Strategy	0.2083	-0.0825	0.4991	1	36	2.5235	0.1209
Order	0.2297	-0.0616	0.5200	1	36	3.1939	0.0823
Strategy:Order	-0.0833	-0.4946	0.3279	1	36	0.1577	0.6936

CI – confidence interval; DF – degrees of freedom; Num – numerator; Den - denominator

Supplemental Digital Content 2
